# Nitric Oxide Stimulates Acute Pancreatitis Pain via Activating the NF-*κ*B Signaling Pathway and Inhibiting the Kappa Opioid Receptor

**DOI:** 10.1155/2020/9230958

**Published:** 2020-05-07

**Authors:** Mengwen Xue, Liang Han, Weikun Qian, Jie Li, Tao Qin, Ying Xiao, Qingyong Ma, Jiguang Ma, Xin Shen

**Affiliations:** ^1^Department of Anesthesiology, The First Affiliated Hospital, Xi'an Jiaotong University, Xi'an 710061, China; ^2^Department of Hepatobiliary Surgery, The First Affiliated Hospital, Xi'an Jiaotong University, Xi'an 710061, China

## Abstract

Pain is the most important clinical feature of acute pancreatitis (AP); however, its specific mechanism is currently unclear. In this study, we showed that AP caused an increase in nitric oxide (NO) secretion, activated the NF-*κ*B pathway in the dorsal root ganglia (DRGs), and caused pain. We established an AP model in vivo and tested the expression of NO, the kappa opioid receptor (KOR), and pain factors. We showed that NO in AP was significantly elevated and increased the expression of pain factors. Next, by treating DRGs in vitro, it was found that NO activated the NF-*κ*B pathway; conversely, NF-*κ*B had no effect on NO. Moreover, inhibition of NF-*κ*B promoted the KOR, whereas NF-*κ*B did not change after KOR activation. Finally, behavioral experiments showed that a NO donor increased the pain behavior of mice, while a NO scavenger, NF-*κ*B inhibitor, or KOR agonist attenuated the pain response in mice. These results suggest that iNOS/NO/NF-*κ*B/KOR may be a key mechanism of pain in AP, providing a theoretical basis for the use of peripheral-restricted KOR agonists for pain treatment in AP.

## 1. Introduction

Acute pancreatitis (AP) is an inflammatory disease that causes pancreatic tissue digestion, edema, hemorrhage, and even necrosis of pancreatic tissue after activation of pancreatic enzyme by multiple causes. Pain is one of the most common clinical symptoms of AP and often causes uncomfortable experiences for patients [1]. Many studies have shown that in inflammatory diseases, the cause of pain involves inflammatory mediators, the nervous system, and the immune system. The interaction between nerves and diseased areas through neurotransmitters and inflammatory mediators affects the progression of pain and disease.

Nitric oxide (NO) is a highly reactive free radical produced by the action of L-arginine through a series of isoenzymes known as nitric oxide synthase (NOS) [2]. NOS is divided into three subtypes, eNOS, iNOS, and nNOS. Among them, iNOS can be expressed in a variety of cells and produce a large amount of NO in a continuous and uncontrolled manner. A growing amount of evidence suggests that a very early event in the development of acute pancreatitis is the release of endogenous inflammatory mediators from the inflamed pancreas. The results of clinical trials and animal models of AP indicate complex interactions between local and systemic inflammatory mediators, including NO [3]. NO is a key signaling molecule in the pathogenesis of inflammation; however, the role of NO in the inflammatory process of AP is still controversial.

Nuclear factor-kappa B (NF-*κ*B) is a protein that specifically binds to the *κ*B site of various cellular genes and initiates transcription of those genes; it is a participation factor of the immune response, inflammation, stress, tissue damage, apoptosis, and so on [4]. NF-*κ*B has been shown to play a key role in the development of acute pancreatitis, and activation of the transcription factor NF-*κ*B can be detected early in experimental pancreatitis [5]. In addition, the activation of the NF-*κ*B-associated pathways mediates IL-1*β*-induced upregulation of spinal COX-2 and pain hypersensitivity following peripheral inflammation [6]. There are various opinions about the relationship between NO and NF-*κ*B. Deletion of the *κ*B site from the NOS2 promoter prevents cytokine-induced increases in NOS2 transcription, which in turn has been found to regulate NF-*κ*B activity in cells [7]. It has been proved that NO represses inhibitory *κ*B kinase through S-nitrosylation [8]. Moreover, NO is an important costimulator of IKK-*α* and NF-*κ*B signaling in endothelial cells; low concentrations of NO can directly act on NF-*κ*B and I*κ*B subunits and can also exert its costimulatory effect by influencing the activity of different known or unidentified signaling molecules that participate in the activation of NF-*κ*B [9].

The innervation of the pancreas consists of intrinsic and extrinsic components. The intrinsic components include myelinated or unmyelinated nerve fibers, thick nerve bundles, and aggregates of neural cell bodies called intrapancreatic ganglia. These ganglionic structures are randomly distributed throughout the pancreatic parenchyma. External nerve fibers can be classified anatomically and functionally into afferent and efferent nerves; the afferent system involved in transmitting sensory signals to the central nervous system (CNS) is composed of thin unmyelinated fibers whose cell bodies are located in the dorsal root ganglia (DRGs) [10]. The kappa opioid receptor (KOR) is an inhibitory G-protein-coupled receptor that is activated by the endogenous ligand dynorphin and is widely expressed in the central and peripheral nervous systems [11]. The current literature found that the NOS/NO pathway is involved in the peripheral antinociceptive effect of KOR [12]. And inhibition of NF-*κ*B signaling could be the mechanism of SNP-mediated KOR gene expression in P19 cells treated with retinoic acid [13].

Pain treatment is a key to conventional treatment of AP. Compared with other analgesic methods, opioids can reduce the need for auxiliary analgesia [14]. Nalbuphine is an opioid which can inhibit the *μ* opioid receptor (MOR) while activating KOR, thus reducing the side effects caused by MOR being excited without affecting its analgesic effect [15].

In summary, we found that NF-*κ*B, iNOS, and NO interact with each other and are closely related and have important significance for AP, but the relationship between these factors and KOR and the relationship between KOR and pain in AP are still unclear. Therefore, we hypothesized that in acute pancreatitis, iNOS can induce NO production and activate the NF-*κ*B signaling pathway, thereby regulating the downstream KOR involved in acute pancreatitis pain, and nalbuphine as a KOR agonist is expected to be an effective analgesic of AP.

## 2. Materials and Methods

### 2.1. Animals and Experimental Protocols

Male C57BL/6 mice weighing 25 g were used for experiments. All mice were housed under pathogen-free conditions and a 12-hour light/dark cycle with free access to water and food. All experimental protocols were approved by the Ethical Committee of the First Affiliated Hospital of Medical College, Xi'an Jiaotong University, Xi'an, China. To induce AP, cerulein (Sigma-Aldrich, St. Louis, MO, USA) was administered by intraperitoneal injection at a dose of 40 *μ*g/kg at 1-hour intervals 6 times a day for 2 days. For the different groups, a NO donor (sodium nitroprusside (SNP), Sigma-Aldrich, St. Louis, MO, USA), NO scavenger (carboxy-PTIO, Beyotime Biotechnology, Shanghai, China), NF-*κ*B inhibitor (PDTC, Beyotime Biotechnology, Shanghai, China), and KOR agonist (nalbuphine, Humanwell Healthcare, Wuhan, China) were administered 1 hour after injection of cerulein.

### 2.2. Extraction and Treatment of Dorsal Root Ganglia

DRGs were isolated from neonatal rats, stored on ice in DMEM : F12 (1 : 1) containing 20% FBS, inoculated into 24-well plates, and fixed with Matrigel. After the Matrigel solidified, DMEM : F12 (1 : 1) was added, and after 24 hours, the medium was replaced by the medium containing SNP, carboxy-PTIO, PDTC, and nalbuphine.

### 2.3. Histology

Pancreatic tissue was fixed in 4% formaldehyde and embedded in paraffin. Hematoxylin & eosin (H&E) staining of fixed pancreatic sections (3 *μ*m thickness) was observed and evaluated under the microscope. For H&E staining, the sections were deparaffinized, stained with hematoxylin for 3 minutes, washed, and restored to blue with a mild alkaline solution. Then, the sections were dehydrated in 85% and 95% ethanol, stained with eosin, and finally dehydrated.

### 2.4. Immunohistochemistry

After deparaffinization of the pancreatic tissue, microwave antigen retrieval was first performed by microwaving the sections in citrate buffer and then incubating in a 3% peroxidase solution (hydrogen peroxide : pure water = 1 : 9). Then, 3% BSA was added dropwise for 30 minutes at room temperature. Primary antibodies were added dropwise to the sections and then incubated overnight at 4°C in a wet box. The sections were then incubated with the secondary antibodies of the corresponding species for 1 hour at room temperature. Finally, the diaminobenzidine tetrahydrochloride substrate was used for color development, counterstained with hematoxylin, and then dehydrated.

### 2.5. Measurement of NO

NO content in the serum of mice was detected by Griess reagent. NO levels were determined by spectrophotometry at 540 nm using a nitric oxide assay kit (Beyotime Biotechnology, Shanghai, China) according to the manufacturer's instructions.

### 2.6. Immunofluorescence

The sections were deparaffinized for antigen retrieval and then blocked in BSA for 30 minutes at room temperature. The sections were incubated with the primary antibody overnight at 4°C. The slides were washed 3 times in PBS for 5 minutes each, and the secondary antibodies of the corresponding species were added dropwise and incubated for 50 minutes at room temperature in the dark. After washing 3 times with PBS, DAPI was added dropwise for nuclear staining and incubated for 10 minutes in the dark. After washing again, the slides were blocked. The primary antibodies used were mouse anti-CGRP (1 : 50, Santa Cruz Biotechnology, CA, USA), mouse anti-substance P (1 : 50, Santa Cruz Biotechnology, CA, USA), and anti-Oprk1 (1 : 50, Sigma-Aldrich, St. Louis, MO, USA).

### 2.7. Western Blot Assay

The proteins were separated by SDS-PAGE, which was preformed according to the instructions, and the separated proteins were transferred to a PVDF membrane. The membrane was blocked with skim milk. After washing with TBS-T, the membrane was incubated with the primary antibody at 4°C overnight. After thorough washing with TBS-T, the secondary antibody was applied to the membrane and detected using a chemiluminescence detection kit and related instruments. The primary antibodies used were mouse anti-CGRP (1 : 500, Santa Cruz Biotechnology, CA, USA), mouse anti-SP (1 : 500, Santa Cruz Biotechnology, CA, USA), anti-Oprk1 (1 : 500, Sigma-Aldrich, St. Louis, MO, USA), rabbit anti-p-P65 (1 : 1000, Cell Signaling Technology, Danvers, MA, USA), rabbit anti-P65 (1 : 1000, Cell Signaling Technology, Danvers, MA, USA), rabbit anti-p-I*κ*B*α* (1 : 1000, Cell Signaling Technology, Danvers, MA, USA), and rabbit anti-I*κ*B*α* (1 : 1000, Cell Signaling Technology, Danvers, MA, USA).

### 2.8. Enzyme-Linked Immunosorbent Assay

Blood samples were collected from the mice and centrifuged at 3000 rpm for 15 minutes at 4°C. The supernatant was suctioned, and the serum was kept at -80°C for further analysis. Next, the expression level of pain factors was determined according to the instructions of the ELISA kit (R&D, Minneapolis, MN, USA).

### 2.9. Real-Time PCR

Total RNA in the pancreas and DRGs was extracted using TRIzol (Invitrogen, CA, USA), and cDNA was synthesized using the PrimeScript RT kit (TaKaRa, Dalian, China). Real-time experiments were performed on an iQ5 multicolor real-time PCR detection system (Bio-Rad, Hercules, CA) using SYBR Green real-time PCR master mix (TaKaRa, CA, USA). Primers for SYBR Green RT-qPCR are shown in Table 1.

### 2.10. Measurements of Pain Behaviors

#### 2.10.1. Twisting Experiment

The mice were placed in a plastic container and allowed to acclimate for 30 minutes. After intraperitoneal injection of cerulein and various intervention agents, the behavior of the mice was observed and videotaped, and the number of writhing reactions in the mice during thirty minutes was counted. The writhing reaction was defined as abdominal contraction, stretching of the trunk and hind legs, lifting of the buttocks, or body twisting.

#### 2.10.2. Von Frey Filament Test (vFF)

Following the protocols of Atsufumi et al. [16], the mice were placed on the wire mesh floor under a transparent plastic box and acclimatized for 30 minutes prior to testing, and the upper abdomen was stimulated with three different filaments in ascending order of strength. The mechanical stimulation of each filament was applied five times at 10-second intervals, and after one minute of rest, it was applied five more times in the same manner. The scores for mouse behavior were defined as follows: score 0 = no response; score 1 = immediately escaped or stunned/scratched; and score 2 = contracted the abdomen or jumped. Data are expressed as the total score of the response caused by 10 challenges with each hair.

#### 2.10.3. Electromyography (EMG)

The mice were fixed on the table under anesthesia. After the skin of the mouse was prepared and disinfected, the skin was exposed and the rectus abdominis muscle was exposed. One end of the electrode was placed in the rectus abdominis muscle, the two electrodes were 1 cm apart, and the other end was connected to a signal acquisition system. At the same time, an electrically stimulating electrode was placed in the rectus abdominis. After the mice were acclimated for 10 minutes, 10 repetitions of 0.05 V string stimulation were given, and the EMG was collected to analyze the changes in EMG amplitude.

### 2.11. Drug Treatments

In vivo drug treatments used were SNP at 17.5 *μ*g/kg, carboxy-PTIO at 1 mg/kg, PDTC at 200 mg/kg, and nalbuphine at 10 mg/kg. In vitro drug treatments used were SNP at 100 *μ*mol/L, carboxy-PTIO at 300 *μ*mol/L, PDTC at 100 *μ*mol/L, and nalbuphine at 100 *μ*g/L.

### 2.12. Statistical Analysis

Statistical analysis was performed using the SPSS software package (SPSS 18.0; SPSS Inc., Chicago, IL, USA). All results are shown as mean ± SD, and Student's *t*-test and analysis of variance (ANOVA) were applied. *p* < 0.05 was considered statistically significant. Each experiment was performed at least three times.

## 3. Results

### 3.1. Acute Pancreatitis Promotes the Expression of NO and iNOS

**Figure 1:**
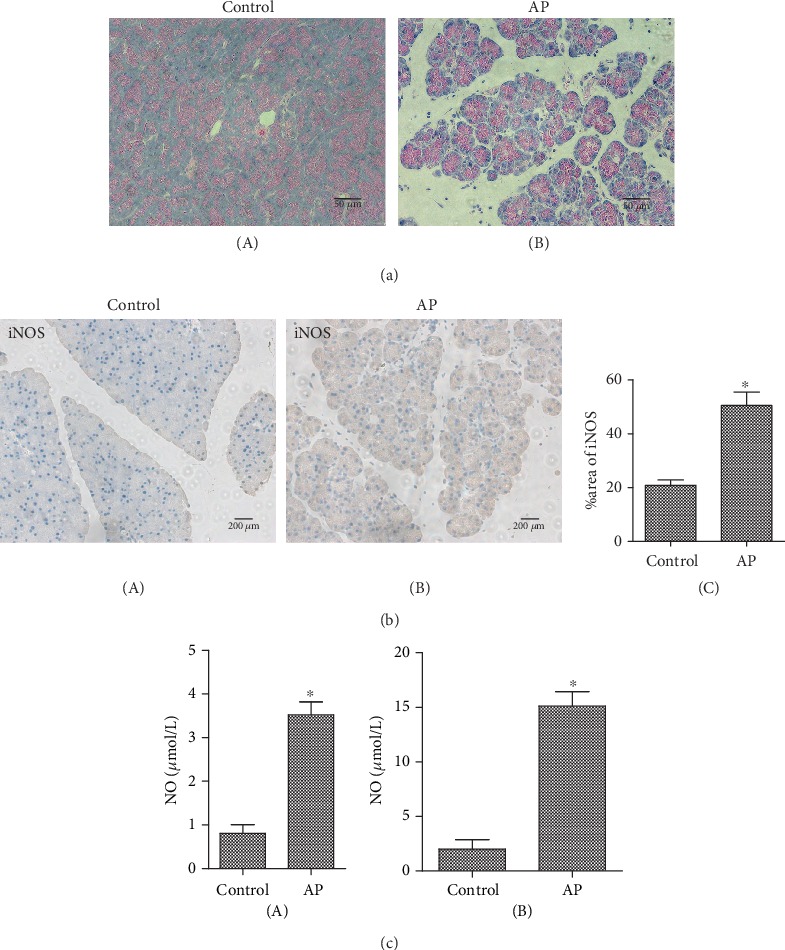
Acute pancreatitis promotes the expression of NO and iNOS. (a) H&E staining of pancreatic tissue in vehicle- or cerulein-treated (40 *μ*g/kg in 100 *μ*L) C57BL/6 mice. Scale bars: 50 *μ*m. (b) Immunohistochemical staining of pancreatic iNOS in control mice (A) or acute pancreatitis mice (B). Scale bars: 200 *μ*m. The experiment was repeated three times. The data were analyzed using Student's *t*-test and one-way ANOVA. ^∗^*p* < 0.05, *n* = 6/group. (c) The NO content in the control group or acute pancreatitis group. A: the serum of mice; B: pancreatic tissues of mice. The experiment was repeated three times. The data were analyzed using Student's *t*-test and one-way ANOVA. ^∗^*p* < 0.05, *n* = 6/group.

To establish the AP model, C57BL/6 mice received intraperitoneal injections of either cerulein (40 *μ*g/kg in 100 *μ*L) or vehicle (PBS) hourly, 6 times a day for two days. H&E staining showed significant edema and a large amount of inflammatory cell infiltration compared to those of the control group (Figure 1(a)). Immunohistochemistry confirmed that the protein expression of iNOS was increased in AP (Figure 1(b)). Similarly, the level of NO in the serum (Figure 1(c), A) and pancreatic tissue (Figure 1(c), B) of mice with AP increased markedly. The above results indicate that AP can cause an increase in the expression of iNOS and produce a large amount of NO.

### 3.2. Acute Pancreatitis Increases the Level of SP and CGRP While Reducing KOR Levels

**Figure 2:**
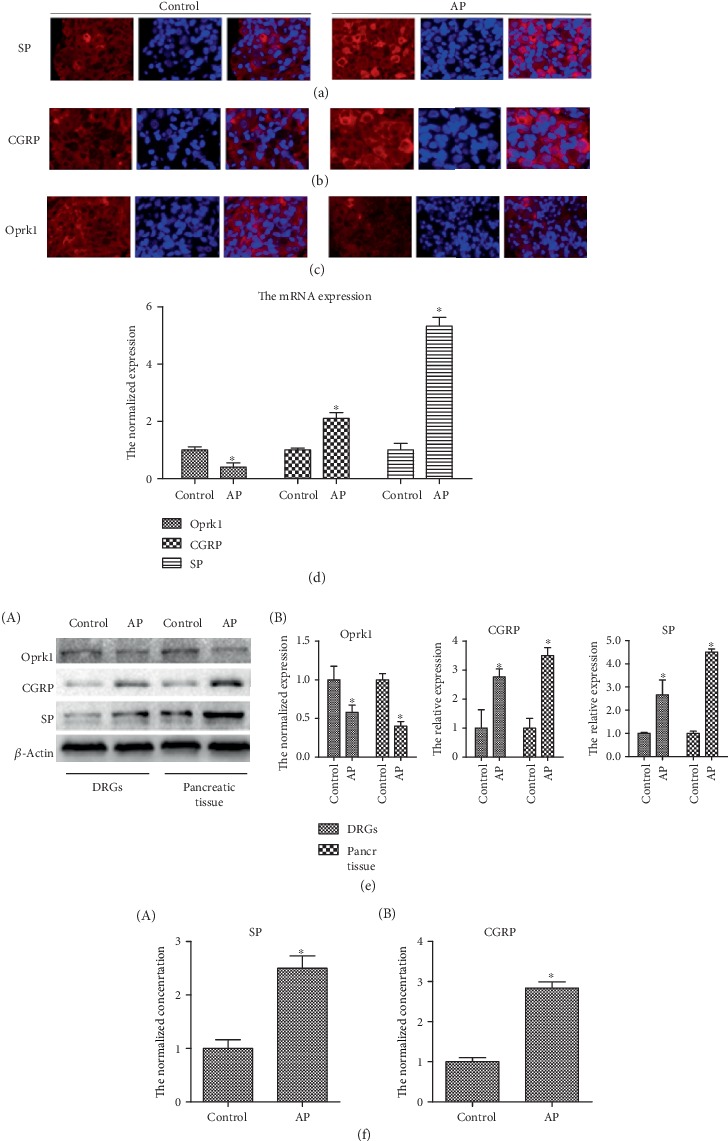
Acute pancreatitis increases the level of SP and CGRP while reducing KOR levels. (a–c) Immunofluorescence staining of DRG sections of mice with or without acute pancreatitis. SP, CGRP, and Oprk1 are stained red, and the nuclei are stained blue. (d) The mRNA expression of Oprk1, SP, and CGRP in DRGs of mice with or without acute pancreatitis. The experiment was repeated three times. The data were analyzed using Student's *t*-test and one-way ANOVA and are presented as the normalized expression relative to the control group. ^∗^*p* < 0.05, *n* = 6/group. (e) Protein expression of Oprk1, SP, and CGRP in DRGs and pancreatic tissue of mice in the control group and acute pancreatitis group as detected by western blot assay. A: gray values were detected by ImageJ (National Institutes of Health), and the data were analyzed using Student's *t*-test and one-way ANOVA and are presented as the normalized expression relative to the control group. ^∗^*p* < 0.05, *n* = 3/group. (f) SP and CGRP levels in the serum of mice in the control group and acute pancreatitis group as detected by the ELISA kit. The data were analyzed using Student's *t*-test and one-way ANOVA and are presented as the normalized expression relative to the control group. ^∗^*p* < 0.05, *n* = 6/group.

Pain is the most typical symptom of AP; therefore, immunofluorescence staining, ELISA, real-time PCR, and western blot analysis were performed to detect changes in KOR, SP, and CGRP levels in DRGs or pancreatic tissue. Under the influence of AP, the levels of SP and CGRP increased significantly, while the expression level of the Oprk1 gene, which encodes the KOR, decreased (Figures 2(a)–2(c), 2(e), and 2(f)). Moreover, the RNA levels of SP and CGRP increased sharply in mice with AP, and the RNA level of Oprk1 decreased less than half of that of the control group (Figure 2(d)), indicating that AP observably causes pain.

### 3.3. NO Promotes the Expression of Pain Factors in Mice with AP

**Figure 3:**
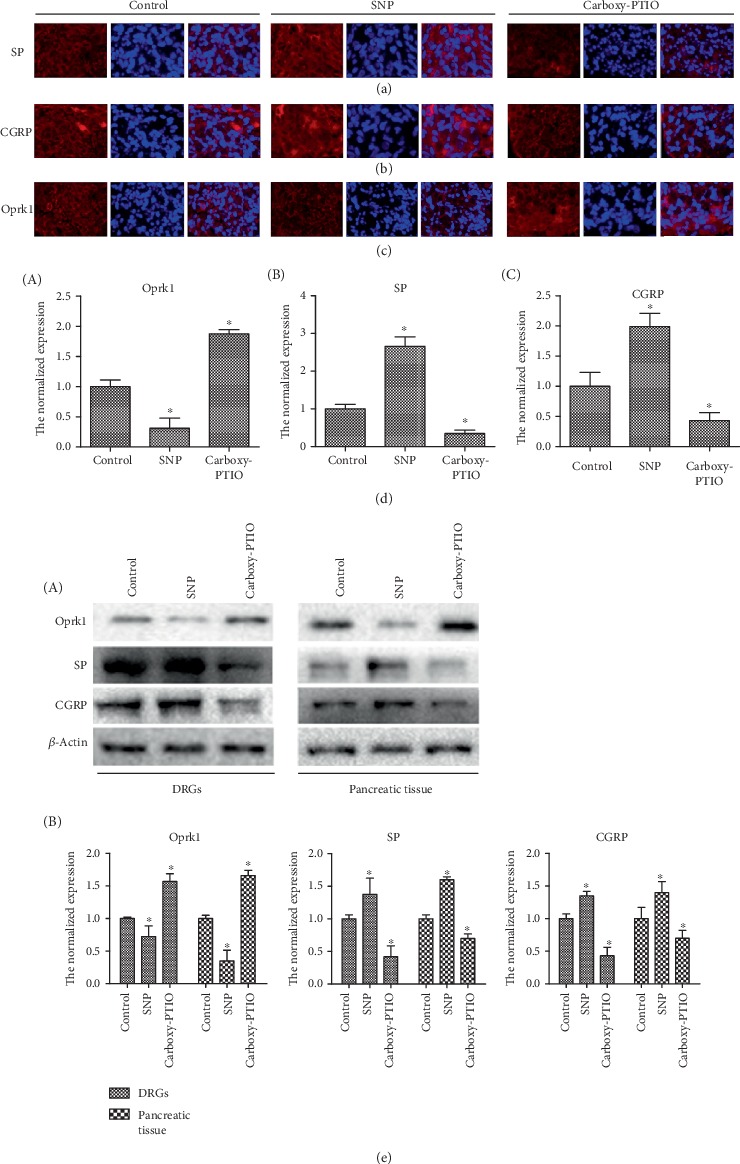
NO promotes the expression of pain factors in mice with acute pancreatitis. (a–c) Immunofluorescence staining of DRG sections of mice with acute pancreatitis treated with the NO donor and scavenger. SP, CGRP, and Oprk1 are stained red, the nuclei are stained blue. (d) The mRNA expression of Oprk1, SP, and CGRP in DRGs of mice with acute pancreatitis treated with the NO donor and scavenger. The experiment was repeated three times. The data were analyzed using Student's *t*-test and one-way ANOVA and are presented as the normalized expression relative to the control group. ^∗^*p* < 0.05, *n* = 6/group. (e) Protein expression of Oprk1, SP, and CGRP in DRGs and pancreatic tissue of mice with acute pancreatitis treated with the NO donor and scavenger as detected by western blot assay. Gray values were detected by ImageJ (National Institutes of Health), and the data were analyzed using Student's *t*-test and one-way ANOVA and are presented as the normalized expression relative to the control group. ^∗^*p* < 0.05. This part of the experiment was carried out in mice with acute pancreatitis.

To explore the role of NO in AP, we used a NO donor (sodium nitroprusside (SNP)) and scavenger (carboxy-PTIO) to treat in AP mice. Immunofluorescence staining showed that compared with the levels in the control group, SP and CGRP in the DRGs of mice increased significantly after SNP intervention (Figures 3(a) and 3(b)), and the expression of Oprk1 decreased notably (Figure 3(c)). Conversely, the expression of SP and CGRP decreased after carboxy-PTIO treatment (Figures 3(a) and 3(b)), and the expression of Oprk1 increased (Figure 3(c)). Western blot analysis results were consistent with the immunofluorescence staining results in both DRGs and pancreatic tissue (Figure 3(e)). Real-time PCR also showed that RNA levels of SP and CGRP increased due to elevated NO, which decreased due to the clearance of NO, while the change in the RNA level of KOR was reversed (Figure 3(d)). These results indicate that NO plays an important role in the pain in AP and that the pain increases with the increase of NO. Furthermore, NO may be able to regulate the KOR in some way.

### 3.4. Molecular Mechanism by Which NO Promotes Pain

**Figure 4:**
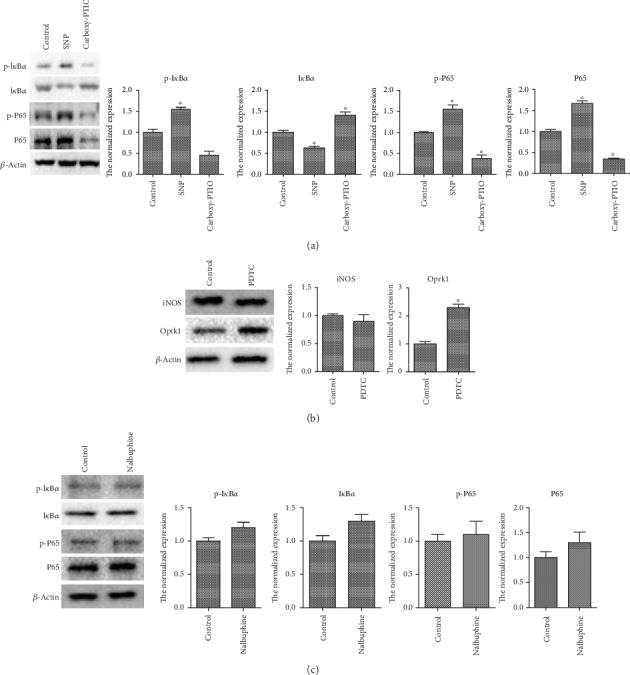
Molecular mechanism by which NO promotes pain. (a) In vitro administration of DRGs with the NO donor and scavenger and western blot assay for the expression of NF-*κ*B pathway-associated proteins. (b) In vitro administration of DRGs with the NF-*κ*B pathway inhibitor and western blot assay for the expression of iNOS and Oprk1. (c) In vitro administration of DRGs with the KOR agonist and western blot assay for the expression of NF-*κ*B pathway-associated proteins. Gray values were detected by ImageJ (National Institutes of Health), and the data were analyzed using Student's *t*-test and one-way ANOVA and are presented as the normalized expression relative to the control group. ^∗^*p* < 0.05.

Previous studies have suggested that the NF-*κ*B signaling pathway is closely related to AP, but its role in AP-associated pain is still unclear. Therefore, we administered SNP and carboxy-PTIO to DRGs in vitro, and then western blot was used to detect the expression of proteins of the NF-*κ*B pathway. The results showed that phosphorylation of I*κ*B*α* increased after SNP intervention, promoting the degradation of I*κ*B*α*, and carboxy-PTIO inhibited I*κ*B*α* phosphorylation and its degradation. Moreover, phosphorylation of P65 and its expression increased with the SNP intervention and reduced after the carboxy-PTIO intervention (Figure 4(a)). These results indicate that NO may promote the activation of the NF-*κ*B signaling pathway and that the NF-*κ*B pathway is also significantly inhibited after removing NO. However, there was no significant difference in iNOS expression after intervention with the NF-*κ*B pathway inhibitor PDTC in DRGs (Figure 4(b)). Based on this, we hypothesize that in AP-associated pain, iNOS/NO is located upstream of NF-*κ*B. Next, we found that Oprk1 expression increased after PDTC intervention (Figure 4(b)). Conversely, after treatment of DRGs with the KOR agonist nalbuphine, there was no significant change in the related proteins of the NF-*κ*B signaling pathway (Figure 4(c)). These results indicate that NF-*κ*B may cause an increase in Oprk1 expression, while changes in Oprk1 do not affect the NF-*κ*B pathway, possibly demonstrating that NF-*κ*B is located upstream of the KOR.

### 3.5. NO Increases the Expression of Pain Factors through NF-*κ*B

**Figure 5:**
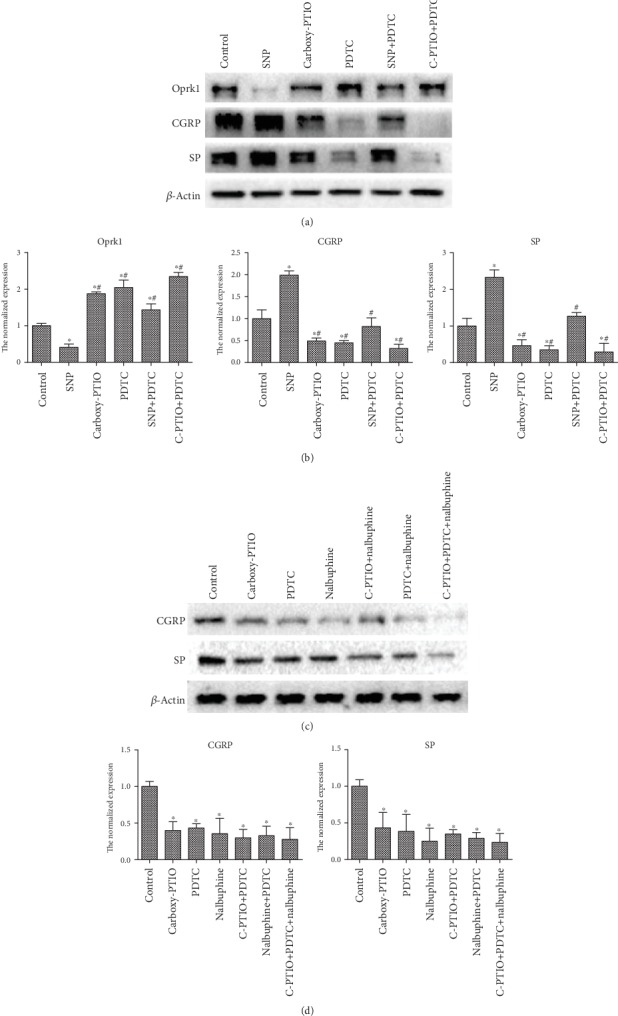
NO increases the expression of pain factors through NF-*κ*B. (a) Protein expression of Oprk1, SP, and CGRP in DRGs treated with the NO donor, scavenger, and NF-*κ*B pathway inhibitor alone or combined as detected by western blot assay. (b) The mRNA expression of Oprk1, SP, and CGRP in DRGs treated with the NO donor, scavenger, and NF-*κ*B pathway inhibitor alone or combined. The experiment was repeated three times. The data were analyzed using Student's *t*-test and one-way ANOVA and are presented as the normalized expression relative to the control group. ^∗^*p* < 0.05 compared to the control group, ^#^*p* < 0.05 compared to the SNP group. (c) Protein expression of SP and CGRP in DRGs treated with the NO scavenger, NF-*κ*B pathway inhibitor, and KOR agonist alone or combined as detected by western blot assay. (d) The mRNA expression of SP and CGRP in DRGs treated with the NO scavenger, NF-*κ*B pathway inhibitor, and KOR agonist alone or combined. The experiment was repeated three times. The data were analyzed using Student's *t*-test and one-way ANOVA and are presented as the normalized expression relative to the control group. ^∗^*p* < 0.05.

To further investigate the role of the NF-*κ*B pathway in the pain of AP, we treated DRGs with SNP, carboxy-PTIO, and the NF-*κ*B pathway inhibitor PDTC alone or together, and western blot and real-time PCR showed that after SNP and carboxy-PTIO intervention, the changes in Oprk1, SP, and CGRP were consistent with the results of the in vivo experiments. PDTC observably promoted the expression of Oprk1 and inhibited the expression of CGRP and SP. After the combined treatment with SNP and PDTC, the expression of Oprk1 decreased slightly. There was a slight increase in the expression of SP and CGRP when SNP was used alone, whereas the combination of carboxy-PTIO and PDTC significantly decreased SP and CGRP (Figures 5(a) and 5(b)). Next, we treated DRGs with carboxy-PTIO, PDTC, and the KOR agonist nalbuphine, individually or combined, and the results showed that compared with the levels in the control group, the pain factors SP and CGRP had different degrees of reduction, and after combined intervention, the reduction was more obvious (Figures 5(c) and 5(d)). Taken together, these results support the idea that NO may regulate the KOR through the NF-*κ*B signaling pathway, which may affect the expression of pain factors.

### 3.6. Effects of NO, NF-*κ*B, and KOR on Pain Behaviors in Mice

**Figure 6:**
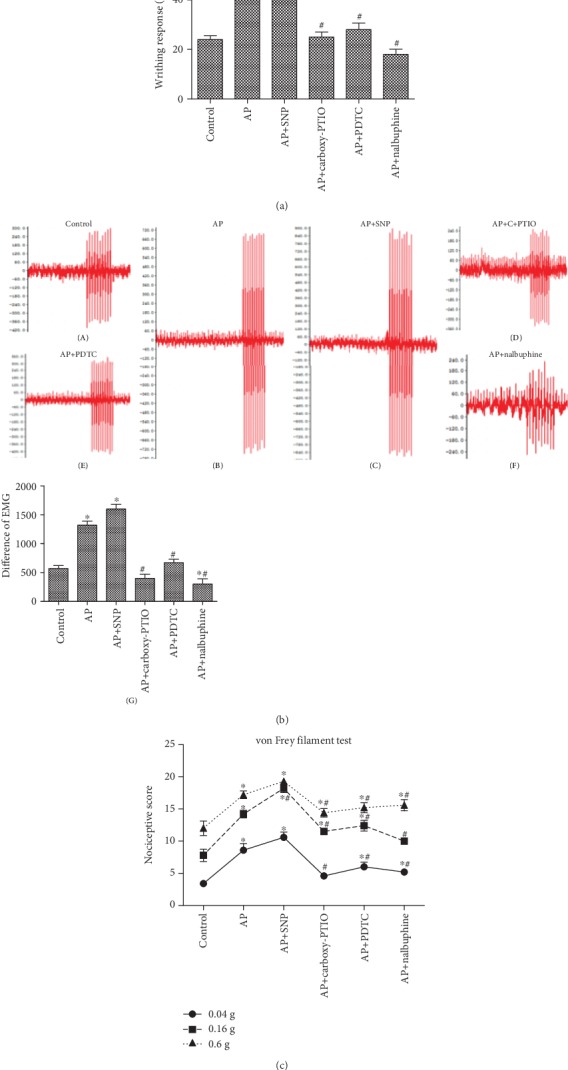
Effects of NO, NF-*κ*B, and the KOR on pain behaviors in mice. (a) The writhing response of mice under different intervention conditions. The writhing response of mice was recorded within 30 minutes, and the experiment was repeated three times. The data were analyzed using Student's *t*-test and one-way ANOVA. ^∗^*p* < 0.05 compared to the control group, ^#^*p* < 0.05 compared to the AP group, *n* = 6/group. (b) The electromyography of mice under different intervention conditions. String stimulation was given 10 times at 0.05 V, and the electromyography was collected to analyze the changes in electromyography amplitude. The experiment was repeated three times, calculating the difference in electromyography amplitude, and the data were analyzed using Student's *t*-test and one-way ANOVA. ^∗^*p* < 0.05 compared to the control group, ^#^*p* < 0.05 compared to the AP group, *n* = 6/group. (c) The von Frey filament test of mice under different intervention conditions. The upper abdomen was stimulated 10 times with filaments of 0.04 g, 0.16 g, and 0.6 g. The data are expressed as the total score of the response caused by 10 repeated challenges with each hair and were analyzed using Student's *t*-test and one-way ANOVA. ^∗^*p* < 0.05 compared to the control group, ^#^*p* < 0.05 compared to the AP group, *n* = 6/group.

To characterize the effects of NO, NF-*κ*B, and KOR on behavior in mice with AP, pain behaviors were assessed by using the established behavioral assays, such as writhing response tests, von Frey behavioral tests, and electromyography experiments. Through the writhing response, we observed that NO significantly increased the pain of the mice, when the NF-*κ*B pathway was inhibited, the pain response was also decreased, and after the KOR was activated, the pain was significantly reduced (Figure 6(a)). In the EMG test, we found that the pain sensitivity of mice with AP increased, and the amplitude of EMG was similarly increased. Treatment with SNP also enhanced this phenomenon, while carboxy-PTIO, PDTC, and nalbuphine significantly reduced the amplitude of myoelectricity in mice with AP and diminished the sensitivity of the mice to pain; the change in the nalbuphine group was even lower than that in the control group (Figure 6(b)). In the VFF test, repeated application of cerulein significantly increased the nociceptive scores in response to mechanical stimulation of different strengths in mice with AP. After SNP intervention, the mice showed a strong enhancement of the score in response to 0.16 g stimulation, while the other three interventions alleviated the nociceptive scores of mice to different degrees of stimulation. Among them, there was no significant difference in the stimulation of 0.16 g in the nalbuphine group and in the stimulation of 0.04 g in the carboxy-PTIO group compared with the control group (Figure 6(c)).

## 4. Discussion

Understanding the specific mechanisms of pain in AP is important because this insight may help in the clinical treatment of AP. The current literature on pain in AP mainly focuses on pancreatic tissue. This study focused on the nerves that innervate the pancreas to explore the pain of pancreatitis at the neurological level. In the present study, we used the in vivo cerulein to establish an AP model and in vitro treatment of DRGs to explore the effects of the peripheral nervous system on pancreatitis pain. The relationship between AP and NO is very close but controversial. Chou et al. found that AP could cause an increase in the expression of iNOS and NO in lung tissue [17]. However, some studies have found that cerulein induced a decrease in NOx levels in the pancreas of rats [18]. Moreover, iNOS is associated with many inflammatory diseases, such as septic shock, rheumatoid arthritis, asthma, and AP [2]. We first tested the levels of iNOS and NO in pancreatic tissue and the NO level in serum from mice with AP and found that the levels of iNOS and NO were significantly increased, which suggested that AP could activate iNOS and induce a large amount of NO secretion.

The sensory nerve is a kind of nerve that conducts afferent impulses from the periphery receptors to the CNS and is capable of releasing neuromediators from the activated peripheral endings. Experimental studies and tissue analysis of pancreatitis have shown significant changes in the sensory nerves that supply the pancreas. The function of primary neurons is to receive information from the external environment and transmit it to the CNS, and activation of these neurons can cause the release of neurotransmitters from peripheral endings [10].

Pain relief is an important part of the treatment of pancreatitis. Compared with other analgesic methods, opioids can reduce the need for auxiliary analgesia, and there is no difference in the risk of pancreatitis complications or clinically severe adverse events between opioids and other analgesics [14]. Therefore, the importance of opioids to pancreatitis is beyond words. Also, opioid receptors have been the focus of decades of research to develop better treatments for pain. During inflammatory processes, opioid receptors in DRGs are transported towards the peripheral sensory nerve endings at the site of inflammation [19]. There are four types of opioid receptors, namely, *μ* (MORs), *δ* (DORs), *κ* (KOR), and nociceptin (NOR). Among them, the KOR is receiving increasing attention. The KOR belongs to the G-protein-coupled seven-transmembrane class of receptors that have widespread distribution in the central and peripheral nervous systems. Opioids can be classified by their actions: agonist, partial agonist, agonist-antagonist, and antagonist. As a selective KOR agonist, nalbuphine antagonizes MOR while activating KOR. Chaim et al. have shown that nalbuphine plays an analgesic role through activating KOR [20]. Existing literature data showed that nalbuphine has no significant difference in pain relief compared to morphine [15] and is more effective for visceral pain [21]. At the same time, nalbuphine can inhibit MOR, thereby reducing nausea, vomiting, itching, and other side effects caused by excited MOR, especially for respiratory suppression, due to the “ceiling effect” of nalbuphine on the respiratory system, which means that its depression effect on the respiratory system at a low dose and breathing is not further compromised with higher doses [21]. A study found that KOR-deficient mice exhibited a dramatic increase in response to visceral pain stimulation compared with the wild-type mice. This suggests that KOR is involved in the perception of visceral pain [22]. In our study, we found that the expression of the KOR was significantly reduced in AP, and the levels of SP and CGRP, which reflect pain, increased, suggesting that AP can affect the expression of the KOR while causing intense pain.

NO is a small, diffusible free radical that acts as a second messenger in the human body. Therefore, to explore the role of NO in pain in AP, we used a NO donor and scavenger to treat mice with AP. When the NO content increased, the expression of Oprk1, which encodes the KOR, decreased, and the pain behavior and pain factor increased significantly. Another interesting finding was when the NO content rose, the NF-*κ*B signaling pathway was activated, and when NO was removed, the NF-*κ*B pathway was also inhibited. It is worth noting that NO has been reported to stimulate and inhibit the activation of the NF-*κ*B pathway in different studies. Mirza et al. found that in macrophages, NOS1-derived NO production leads to proteolysis of suppressor of cytokine signal 1 (SOCS1) and alleviates its repression of NF-*κ*B transcriptional activity [23]. In contrast, Reynaert et al. found that NO possesses an anti-inflammatory effect that may be exerted via its ability to inhibit the transcription factor NF-*κ*B [8]. In addition, Umansky et al. studied the effects of various concentrations of NO on the activation of the transcription factor NF-*κ*B and found that low concentrations of NO enhanced the activation of NF-*κ*B, while high doses of NO impaired the TNF-*α*-inducted DNA-binding activity of NF-*κ*B [9]. However, previous studies have revealed that the NF-*κ*B pathway is activated in AP, which is consistent with the results of this study; AP can cause an increase in NO secretion; and then the increase in NO activates the NF-*κ*B pathway. Therefore, we inhibited the NF-*κ*B pathway in DRGs and found that the expression of Oprk1 was upregulated and expressions of SP and CGRP were inhibited, demonstrating that NF-*κ*B can promote pain. At the same time, we found that iNOS and NO did not change significantly after NF-*κ*B was inhibited, and the KOR expression increased, while KOR agonists did not cause changes in proteins of the NF-*κ*B pathway, which proved that NF-*κ*B may be located downstream of iNOS and upstream of the KOR.

Based on these results, a NO donor and scavenger in combination with NF-*κ*B were used to treat DRGs. Not surprisingly, the results showed that when SNP and carboxy-PTIO were combined with PDTC, changes in Oprk1, SP, and CGRP were intermediate between the separate interventions. The above results indicate that NO and NF-*κ*B act together to affect pain in AP. Moreover, the NF-*κ*B pathway can change with a change in NO content, and so we hypothesize that NO can activate the NF-*κ*B signaling pathway, thereby affecting the expression of the KOR and pain in AP. Finally, we used the selective KOR agonist nalbuphine to treat the DRGs, individually or in combination with other intervention agents, and SP and CGRP were reduced to varying degrees.

As a typical clinical symptom of AP, pain is extremely important for diagnosis and treatment and has always been the focus of research. The DRGs are primary neurons, and their distal ends are distributed in various tissues and organs. DRGs are responsible for receiving all the nerve impulses from the body receptors and transmitting them to the spinal cord. In the diseases of the pancreas, there are nociceptive, neuropathic, and inflammatory components of pain; among them, neuropathic pain is caused by damage to nerve endings within the pancreas and by changes in the neural plasticity of the peripheral nervous system and the central nervous system [24]. Our work builds on these studies, and for the first time, we focused on the nerves that innervate the pancreas. Through our study, we hypothesized that in AP, iNOS-derived NO activates the NF-*κ*B pathway in DRGs and inhibits the expression of the KOR, resulting in the massive release of pain factors. Opioids have attracted much attention as part of the conventional treatment of AP, and selective KOR agonists have emerged as a hot spot of pain research in recent years; they have the advantages of excellent analgesic effects and few side effects. Importantly, Barlass et al. found that morphine increased the severity of AP via MOR [25]. Moreover, according to our findings, the KOR is an important component of pain in AP. As a KOR agonist/MOR antagonist, nalbuphine retains analgesic effect while reducing the adverse reactions caused by excited MOR. Therefore, nalbuphine may be a novel and promising therapeutic approach to reduce pain in AP.

## Figures and Tables

**Table 1:** Real-time PCR primer sequence.

Gene	Primer sequence
Oprk1	P1: 5′-CCGATACACGAAGATGAAGAC-3′ P2: 5′-GTGCCTCCAAGGACTATCGC-3′
SP	P1: 5′-GACTCCTCTGACCGCTAC-3′ P2: 5′-AGACCTGCTGGATGAACT-3′
CGRP	P1: 5′-AACCTTAGAAAGCAGCCCAGGCATG-3′ P2: 5′-GTGGGCACAAAGTTGTCCTTCACCA-3′
*β*-Actin	P1: 5′-CCGTTCCGAAAGTTGCCTTTT-3′ P2: 5′-GAGGCGTACAGGGATAGCAC-3′

## Data Availability

The data that support the findings of this study are available from the corresponding author upon reasonable request.
